# Local Use of Salvarsan

**Published:** 1915-02

**Authors:** 


**Local Use of Salvarsan.** Achard, "Monde Med.," January, reports prompt cure in Vincent's angina, scarlatinal necrotic stomatitis, pyorrhoea alveolaris, leg ulcers, chancroid and phagedena, quoting various authorities.

